# Acute Toxic Injuries of Rat’s Visceral Tissues Induced by Different Oximes

**DOI:** 10.1038/s41598-019-52768-4

**Published:** 2019-11-11

**Authors:** Vesna Jaćević, Eugenie Nepovimova, Kamil Kuča

**Affiliations:** 1 National Poison Control Centre, Military Medical Academy, Belgrade, Serbia; 2 Faculty of Medicine of the Military Medical Academy, University of Defense, Belgrade, Serbia; 3 Department of Chemistry, Faculty of Science, University of Hradec Kralove, Hradec Kralove, Czechia

**Keywords:** Health care, Health occupations

## Abstract

Certain AChE reactivators, asoxime, obidoxime, K027, K048, and K075, when taken in overdoses and sometimes even when introduced within therapeutic ranges, may injure the different organs. As a continuation of previously published data, in this study, Wistar rats have sacrificed 24 hrs and 7 days after single *im* application of 0.1LD_50_, 0.5LD_50_ and 1.0LD_50_ of each reactivator, and examinated tissue samples were obtained for pathohistological and semiquantitative analysis. A severity of tissue alteration, expressed as different tissue damage scores were evaluated. Morphological structure of examinated tissues treated with of 0.1LD_50_ of all reactivators was comparable with the control group of rats. Moderate injuries were seen in visceral tissues treated with 0.5LD_50_ of asoxime, obidoxime and K027. Acute damages were enlarged after treatment with 0.5LD_50_ and 1.0LD_50_ of all reactivators during the next 7 days. The most prominent changes were seen in rats treated with 1.0LD_50_ of K048 and K075 (P < 0.001 vs. control and asoxime-treated group). All reactivators given by a single, high, unitary dose regimen, have an adverse effect not only on the main visceral tissue, but on the whole rat as well, but the exact mechanism of cellular injury remains to be confirmed in further investigation.

## Introduction

Organophosphorus compounds are broadly used as pesticides in agriculture or as chemical warfare agents in the military attacks. In the last decades, they were misused during a terrorist attack in Japan, Syria or most recently in Malaysia^1–3^. With the growing threat of terrorism, the possible intoxication caused by these agents is relatively high. Due to this, many countries are focused on the development of novel more efficient antidotes against these agents^4,5^.

**Figure 1:**
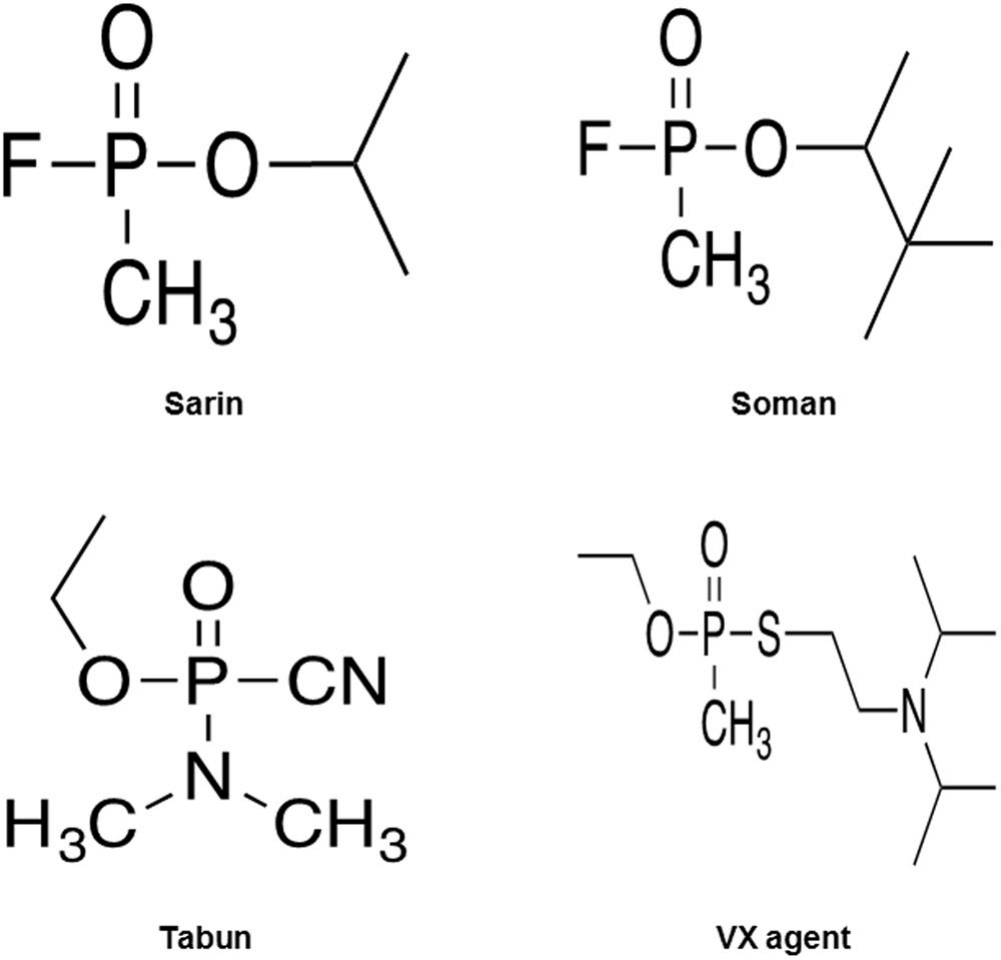
The molecular formula of nerve agents.

Among the most well-known representatives of nerve agents are sarin (GB; O-isopropylmethylfluorophosphate), soman (GD, O-pinacolylmethylfluorophosphate), tabun (GA, O-thyldimethylamidocyanophosphate) and VX (O-ethyl-S- (2-diisopropylaminoethyl) methylthiophosphonate) (Fig. 1).

Their toxic effect is based on the inhibition of enzyme acetylcholinesterase (AChE; EC 3.1.1.7) through its phosphorylation. As a result of this inhibition, this enzyme can not fulfil its physiological function in the organism (cleavage of a neuromediator acetylcholine at nerve synapses). Afterwards, its accumulated amount overstimulates nicotinic and muscarinic receptors. Clinically, it is manifested as a cholinergic crisis. The intoxicated organism then dies without treatment as a result of respiratory and heart failure^6,7^. Standard antidotal treatment of organophosphororus poisoning (OP) implies the administration of anticholinergic drugs to counteract muscarinic over-stimulation, an oxime to reactivate OP-inhibited AChE, and anticonvulsants to protect against central nervous system seizures^8–10^.

**Figure 2:**
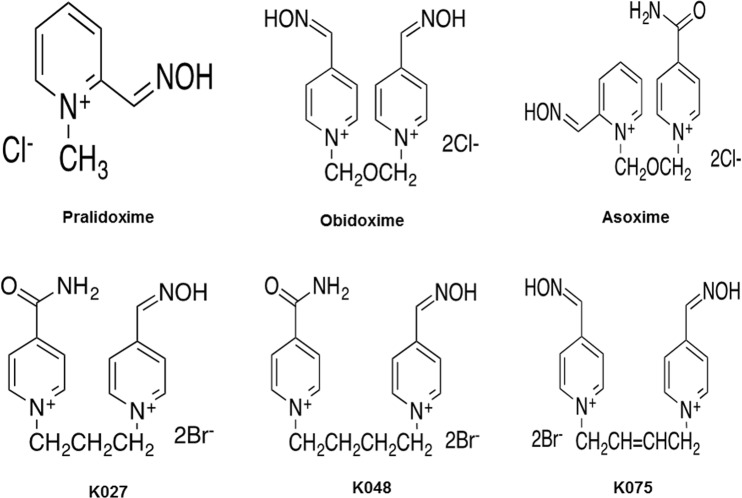
The molecular formula of standard and experimental oxime reactivators.

AChE reactivators are a group of drugs designed to restore AChE function. Among the most well-known representatives of this family are pralidoxime (2-PAM, 2-hydroxyiminomethyl-1-methylpyridinium chloride), obidoxime (Toxogonin, 1,3- bis(4-hydroxyiminomethylpyridinium)-2-oxo-propane dichloride) or asoxime (1-(2-hydroxyiminomethylpyridinium)-3-(4-carbamoylpyridinium)-2-oxapropane dichloride) (Fig. 2).

Their reactivating effect lies in cleavage of the binding resulting from inhibition between the enzyme and the nerve agents^11,12^. None of the so far synthesized AChE reactivators is able to reactivate the enzyme inhibited by all types of nerve agents. That is why many institutions involved in this research area try to predict and then synthesize the structures of an ideal broad-spectrum AChE reactivator that can reactivate the enzyme inhibited by all types of nerve agents^13–17^.

Currently used AChE reactivators are chemically among the mono (pralidoxime) or bisquaternary (obidoxime, asoxime, methoxide) pyridinium compounds with a functional aldoxime group. These substances must contain in their structure some important features without which their reactivation capacity would be low or zero. The most important are: the presence oxime groups (responsible for cleavage of nerve agent-enzyme complex), the presence of quaternary nitrogen (responsible for affinity to AChE and for very good solubility of reactivators), etc.^9,11^.

Although the structure-activity relationship between reactivators’ structure and their biological activity is well known (both reactivation efficacy and inhibition potency), data describing their toxic effect are rare and not complexly investigated^18,19^. If novel reactivators are investigated throughout the world generally their cytotoxicity or acute toxicity is tested^20,21^. Sometimes, their pharmacokinetics with a special focus on blood-brain barrier penetration is investigated^22–24^. However, what is the real cause of the toxicity of reactivators?

Since all the toxicological data were provided in the earlier studies^25,26^, in this third study, we focused our attention on an investigation of morphological lesions of visceral tissue’s produced by increasing doses of selected AChE reactivators. For this purpose, standard oximes (i.e. asoxime and obidoxime) and K-oximes (i.e. K027, K048, and K075) were selected as experimental reactivators. The whole experiment was conducted on Wistar rats.

## Results

### The general health condition of treated rats

In rats treated with 0.1LD_50_ or 0.5LD_50_ dose of different oximes, the clinical signs of acute poisoning were not seen. The muscular pain, weakness, and tremors were perceived only in rats after treatment with 1.0LD_50_ dose of each oxime during the whole study.

### Pathohistological and semiquantitative analysis of experimental animal’s pulmonary alterations

**Figure 3:**
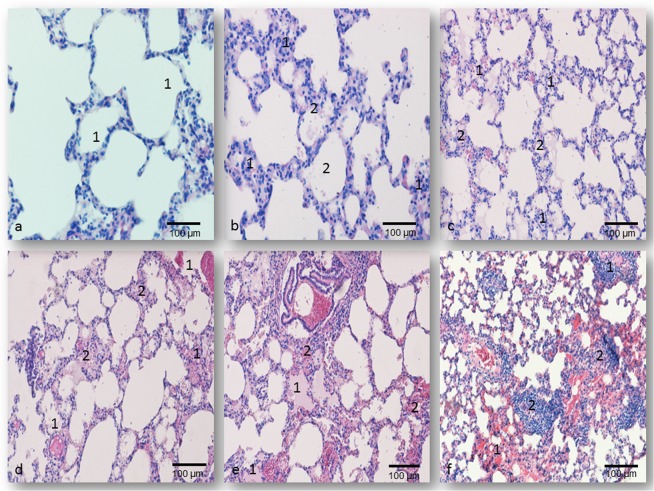
Pulmonary micrographs of rats on day 7 following oximes’ application; H&E staining; magnification at 200× ; (**a**) The normal histological structure of the epithelial cells of control rats - **1**; (**b**) The obidoxime-treated group (0.5LD_50_ *im*), extensive interstitial oedema and hyperemia - **1**, seromucous exudates in alveoli - **2**; (**c**) The K027-treated group (0.5LD_50_ *im*), intensive contraction of alveoli - **1**, diffuse accumulation of inflammatory cells in the pulmonary parenchyma - **2**; (**d**) The obidoxime-treated group (1.0LD_50_ *im*), focal hemorrhage - **1**, interstitial accumulation of inflammatory cells - **2**; (**e**) The K048-treated group (1.0LD_50_ *im*), intra-alveolar - **1**, and interstitial hemorrhage - **2**; (**f**) The K075-treated group (1.0LD_50_ *im*), compressed alveolar space filled with necrotic debris - **1**, interstitial accumulation of macrophages and fibroblasts - **2**.

The lungs’ histological features of rats treated with 0.1LD_50_ dose of all oximes were similar to the control values. A small number of alveoli were filled with desquamated epithelial cells and single alveolar macrophages only in rats than received K075 (Fig. 3a and Table 1).

**Table 1:** The influence of various oximes (0.1LD_50_, 0.5LD_50_ or 1.0LD_50_) on the pulmonary injury (pulmonary damage score, PDS) on day 1 and 7 following application.

Treatments	Days after treatment	PDS (5 lungs/group × 6 specimens/lung) $\bar{{\boldsymbol{\times }}}$ ± S.D.		
		0.1LD_50_	0.5LD_50_	1.0LD_50_
control	1	0.17 ± 0.38	0.17 ± 0.38	0.17 ± 0.38
	7	0.17 ± 0.38	0.17 ± 0.38	0.17 ± 0.38
asoxime	1	0.17 ± 0.38	1.67 ± 0.48**a**^**3**^	2.67 ± 0.48**a**^**3**^
	7	0.20 ± 0.41	2.67 ± 0.48**a**^**3**^**c**^**2**^	2.97 ± 0.69**a**^**3**^
obidoxime	1	0.27 ± 0.45	2.27 ± 0.69**a**^**3**^**b**^**1**^	3.06 ± 0.78**a**^**3**^
	7	0.23 ± 0.45	3.27 ± 0.69**a**^**3**^**c**^**2**^	4.00 ± 0.83**a**^**3**^**b**^**2**^**c**^**2**^
K027	1	0.20 ± 0.41	1.86 ± 0.73**a**^**3**^	2.40 ± 0.50**a**^**3**^
	7	0.23 ± 0.43	2.87 ± 0.73 **a**^**3**^**c**^**3**^	3.57 ± 0.77 **a**^**3**^**c**^**3**^
K048	1	0.40 ± 0.49	2.87 ± 0.73**a**^**3**^**b**^**2**^	4.00 ± 0.83**a**^**3**^**b**^**3**^
	7	0.27 ± 0.45	3.40 ± 0.50**a**^**3**^**c**^**1**^	4.17 ± 0.70**a**^**3**^**b**^**3**^
K075	1	0.33 ± 0.48	3.53 ± 0.51**a**^**3**^**b**^**3**^	4.30 ± 0.70**a**^**3**^**b**^**3**^
	7	0.27 ± 0.45	3.53 ± 0.51**a**^**3**^**b**^**3**^	4.50 ± 0.51**a**^**3**^**b**^**3**^

Statistical evaluation: The Kruskall-Wallis test (between columns), ANOVA test (within columns); **a**^**3**^ - P < 0.001 vs. control group; **b**^**1**^, **b**^**2**^, **b**^**3**^ - P < 0.05, 0.01, 0.001 vs. asoxime-treated group; **c**^**1**^, **c**^**2**^, **c**^**3**^ - P < 0.05, 0.01, 0.001 vs. 1^st^ day.

The pulmonary lesions observed in rats treated with 0.5LD_50_ of each oxime included dilation of lymphatic’s and small blood vessels, early/mild oedema than caused discrete perivascular widening and separation of interstitial tissue, and focal interstitial haemorrhage. In this early phase, 24 hrs after administration, in all oximes treated groups injury of the vascular endothelial cells and the alveolar epithelial cells could be seen. These alterations caused alveolar oedema, which was less intensive in the asoxime-treated rats (PDS value was 1.67 ± 0.48; P < 0.001 vs. control group) and in the K027-treated rats (1.86 ± 0.73; P < 0.001 vs. control group), respectively. On day 7 of the study, the type of histopathological alterations was similar to those established after 24 hrs. The time-dependent differences were seen in all animals treated with asoxime, obidoxime (Fig. 3b) (P < 0.01), or K027 (Fig. 3c) (P < 0.001), while in rats exposed to K048 it was less intensive (P < 0.05). These differences could not be seen after treatment with K075 (PDS values were 3.53 ± 0.51 after day 1 and 7; P < 0.001 vs. asoxime-treated and control groups, respectively) (Table 1).

A single injection of 1.0LD_50_ of different oximes caused severe and diffuse pulmonary alterations which are characteristic of inflammatory interstitial pneumonia. In all treated groups, sacrificed on day 1 of the study, acute interstitial inflammation was characterized by the massive influx of macrophages into alveoli and vacuolisation of their epithelial cells. The vacuolisation of capillary endothelial cells were observed, too. These alterations induced subsequently serofibrinous exudation into alveolar space. Periodically, hyaline membranes lined the alveolar spaces, and numerous leukocytes were infiltrating into the pulmonary parenchyma. In all oximes-treated groups, the pulmonary parenchyma was infiltrated with massive haemorrhagic foci. The strongest PDS values were seen in rats treated along with K048 or K075 (PDS values were 4.00 ± 0.83 or 4.30 ± 0.70; P < 0.001 vs. asoxime-treated rats) (Table 1). Further development of pulmonary injury, in our study on the seventh day, was characterized by the massive intraalveolar aggregation of macrophages, vacuolisation of alveolar cells type II, as well as massive haemorrhages, fibroblast proliferation and collagen deposit in the lung interstitial tissue. The smooth muscle cells of the bronchial wall were the most prominent. These alterations were less intensive in the asoxime-treated rats (PDS value was 2.97 ± 0.69; n.s. vs. the 1^st^ day) and in the K027-treated rats (3.57 ± 0.77; P < 0.001 *vs*. the 1^st^ day), respectively. Noticeable pulmonary injury was established in tissue samples of rats, sacrificed 7 days after treatment with obidoxime, K048 or K075 (Fig. 3d–f), and their PDS values were in the range of 4.17 ± 0.70 to 4.50 ± 0.51, respectively (P < 0.001 vs. control or asoxime-treated rats).

### Pathohistological and semiquantitative analysis of experimental animal’s gastric alterations

**Figure 4:**
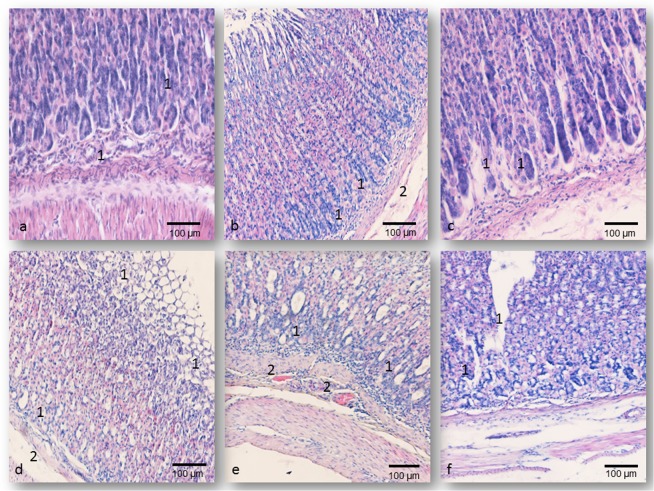
Gastric micrographs of rats on day 7 following oximes’ application; H&E staining; magnification at 200×; (**a**) The normal histological structure of the gastric wall of control rats; (**b**) The K027-treated group (0.5LD_50_ *im*), cellular debris in the bottom of the gastric pits - **1**, oedema and hyperemia in tunica submucosa - **2**; (**c**) The K048-treated group (0.5LD_50_ *im*), degeneration of glandular cells - **1**; (**d**) The K027-treated group (1.0LD_50_ *im*), gastric erosions in the *tunica mucosa* - **1**, enlarged blood vessels and cellular infiltrations in the *tunica submucosa* - **2**; (**e**) The obidoxime-treated group (1.0LD_50_ *im*), enlarged gastric pit filled with necrotic cellular debris - **1**. focal haemorrhages - **2**. (**f**) The K075-treated group (1.0LD_50_ *im*), massive gastric ulcerations - **1**.

In the majority of gastric tissue samples, rats treated with 0.1LD_50_ of each oxime, histological findings were similar to those observed in the control rats (Fig. 4a). However, a small number of tissue sections were shown mild, focal histological changes, such as discrete desquamation of superficial epithelial cells and less intensive mucous fluid. These histological alterations were the highest in rats sacrificed 24 hrs after administration of K075 (GDS was no higher than 0.46 ± 0.61) (Table 2).

**Table 2:** The influence of various oximes (0.1LD_50_, 0.5LD_50_ or 1.0LD_50_) on the gastric injury (gut damage score, GDS) on day 1 and 7 following application.

Treatments	Days after treatment	GDS (5 guts/group × 6 specimens/gut) $\bar{{\boldsymbol{\times }}}$ ± S.D.		
		0.1LD_50_	0.5LD_50_	1.0LD_50_
control	1	0.10 ± 0.31	0.10 ± 0.31	0.10 ± 0.31
	7	0.17 ± 0.38	0.17 ± 0.38	0.17 ± 0.38
asoxime	1	0.13 ± 0.35	1.43 ± 0.97**a**^**3**^	2.13 ± 0.78**a**^**3**^
	7	0.17 ± 0.38	2.33 ± 0.48**a**^**3**^**c**^**2**^	2.63 ± 0.96**a**^**3**^
obidoxime	1	0.33 ± 0.48	2.00 ± 0.64**a**^**3**^**b**^**1**^	2.76 ± 0.77**a**^**3**^**b**^**1**^
	7	0.20 ± 0.41	3.06 ± 0.58**a**^**3**^**c**^**3**^	3.47 ± 1.04**a**^**3**^**c**^**2**^
K027	1	0.17 ± 0.38	1.40 ± 0.50**a**^**3**^	2.68 ± 0.77**a**^**3**^**b**^**1**^
	7	0.20 ± 0.41	2.63 ± 0.96**a**^**3**^**c**^**3**^	3.20 ± 0.76**a**^**3**^**b**^**3**^**c**^**2**^
K048	1	0.37 ± 0.49	2.50 ± 0.51**a**^**3**^**b**^**3**^	3.60 ± 0.97**a**^**3**^**b**^**3**^
	7	0.23 ± 0.43	3.50 ± 0.51**a**^**3**^**c**^**2**^	4.13 ± 0.73**a**^**3**^**c**^**2**^
K075	1	0.23 ± 0.43	2.93 ± 0.78**a**^**3**^**b**^**3**^	4.36 ± 0.72**a**^**3**^**b**^**3**^
	7	0.46 ± 0.61	3.80 ± 0.76**a**^**3**^**b**^**3**^**c**^**2**^	4.53 ± 0.73**a**^**3**^**b**^**3**^

Statistical evaluation: The Kruskall-Wallis test (between columns), ANOVA test (within columns); **b**^**1**^, **b**^**3**^ - P < 0.005, 0.001 vs. asoxime-treated group; **c**^**2**^, **c**^**3**^ - P < 0.01, 0.001 vs. 1^st^ day.

Pathohistological changes exerted in the groups of rats treated by 0.5LD_50_ of each oxime ranged from focal degeneration to necrosis of some epithelial and glandular cells. These histological alterations were associated with moderate vascular changes, such as transmural oedema, massive hyperaemia of the *lamina epithelial tunica mucosae*, and the *tunica submucosa*. Focal, severe haemorrhages were present only in the *tunica submucosa*, predominantly in the perivascular areas. In all oximes-treated groups, various amount of the inflammatory cells (lymphocytes, neutrophils and plasma cells) were accumulated in the vicinity of the blood vessels or in all parts of the *tunica mucosa*. Mild, diffuse oedema and hyperaemia were seen in the *tunica muscularis*. These smooth muscular layers were infiltrated with oedema fluid. In some animals, sacrificed on the day 1, focal necrosis of the epithelial cells was caused a deep defect (gastric erosions) of the *tunica mucosa*, which was extending only into a superficial part of the mucosa. This superficial loss of normal mucosa accompanied by congestion and mild haemorrhages were the highest in rats treated by K048 (GDS = 2.50 ± 0.51), or K075 (GDS = 2.93 ± 0.78). The frequency and severity of these gastric lesions were significantly intensive in comparison to asoxime-treated rats or the control rats, sacrificed after day 1 (P < 0.001). The time-dependent differences were the higher in the K075-treated rats (P < 0.01), and the highest in the asoxime-treated, obidoxime-treated, K027-treated (Fig. 4b) or K048-treated (Fig. 4c) groups (P < 0.001) (Table 2).

Described pathohistological alterations were also seen in the groups of rats treated by 1.0LD_50_ of each oxime, but their intensity was more frequent. The most interesting findings were diffuse necrosis of mucosal epithelial cells which were leading to loss of the mucosal epithelium, especially in the K048 or K075-treated groups, sacrificed after day 1, as well as in the obidoxime-treated animals sacrificed on the day 7 of the study. Consequently, there were presented collapse of the mucosa, focal ulcerations, massive haemorrhage and secondary inflammatory cell infiltration. These defects of the mucosa were extending into the *lamina propria tunica mucosae* of the K027-treated rats (Fig. 4d) (GDS value was up to 3.47 ± 1.04; P < 0.001 vs. asoxime-treated or control groups), or into the *tunica submucosa* of the obidoxime-treated (Fig. 4e) or K075-treated rats (Fig. 4f) (GDS values were in the range of 4.13 ± 0.73 to 4.53 ± 0.73; P < 0.001 vs. asoxime-treated or control groups). These morphological changes were significantly intensive in the group of animals treated with K048 (P < 0.01), obidoxime or K027 (P < 0.05) which were sacrificed on the seventh day of the study (Table 2).

### Pathohistological and semiquantitative analysis of experimental animal’s hepatic alterations

**Figure 5:**
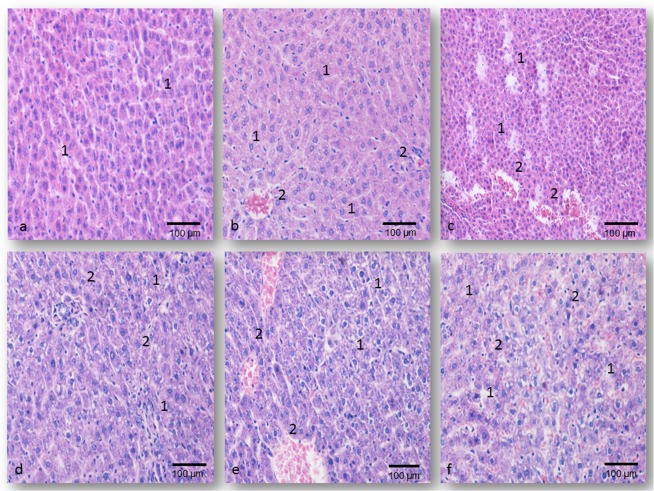
Hepatic micrographs of rats on day 7 days following oximes’ application; H&E staining; magnification at 200×; (**a**) The normal histological structure of the hepatic tissue of control rats - **1**; (**b**) K048-treated group (0.5LD_50_ *im*), micronodular degeneration of the hepatocytes - **1**, and focal hemorrhages - **2**; (**c**) The K075-treated group (0.5LD_50_ *im*), small necrotic foci - **1**, and focal hemorrhages - **2**; (**d**) The asoxime-treated group (1.0LD_50_ *im*), macronodular vacuolization of the hepatocytes - **1**, and moderate hyperemia and oedema - **2**; (**e**) The K027-treated group (1.0LD_50_ *im*), centrolobular necrotic cells - **1**, and focal hemorrhages - **2**; (**f**) The K048-treated group (1.0LD_50_ *im*), multilobular necrosis - **1**, and accumulation of neutrophils, lymphocytes, plasma cells and macrophages - **2**.

In rats treated by 0.1LD_50_ of each oxime, isolated hepatocytes with intracytoplasmatic vacuoles were seen in central lobular areas. In these hepatic sections, mild oedema and hyperemia, as a result of the blood vessels dilatation, were presented in the sinusoids. Also, the extravascular cell infiltrate was observed in rats than treated by obidoxime, K048 or K075. In these groups of rats, sacrificed after day 1, the HDS values were in the range of 0.27 ± 0.45 to 0.33 ± 0.48, and similar to other treated rat groups and control group (Fig. 5a and Table 3).

**Table 3:** The influence of various oximes (0.1LD_50_, 0.5LD_50_ or 1.0LD_50_) on the hepatic injury (hepatic damage score, HDS) on day 1 and 7 following application.

Treatments	Days after treatment	HDS (5 livers/group x 6 specimens/liver) $\bar{{\boldsymbol{\times }}}$ ± S.D.		
		0.1LD_50_	0.5LD_50_	1.0LD_50_
control	1	0.20 ± 0.41	0.20 ± 0.41	0.20 ± 0.41
	7	0.17 ± 0.38	0.17 ± 0.38	0.17 ± 0.38
asoxime	1	0.20 ± 0.41	1.90 ± 0.61**a**^**3**^	2.70 ± 0.70**a**^**3**^
	7	0.20 ± 0.41	2.67 ± 0.48**a**^**3**^**c**^**2**^	2.73 ± 0.69**a**^**3**^
obidoxime	1	0.33 ± 0.48	2.73 ± 0.69**a**^**3**^**b**^**2**^	3.26 ± 0.69**a**^**3**^**b**^**1**^
	7	0.20 ± 0.41	3.13 ± 0.51**a**^**3**^**c**^**1**^	3.50 ± 0.73**a**^**3**^
K027	1	0.20 ± 0.41	2.10 ± 0.76 **a**^**3**^	2.83 ± 0.79**a**^**3**^
	7	0.27 ± 0.45	3.07 ± 0.45**a**^**3**^**c**^**2**^	3.43 ± 0.50**a**^**3**^**c**^**2**^
K048	1	0.27 ± 0.45	2.93 ± 0.87**a**^**3**^**b**^**3**^	3.87 ± 0.78**a**^**3**^**b**^**3**^
	7	0.20 ± 0.41	3.87 ± 0.78**a**^**3**^**c**^**2**^	4.33 ± 0.53**a**^**3**^**b**^**3**^**c**^**1**^
K075	1	0.33 ± 0.48	3.06 ± 0.78**a**^**3**^**b**^**3**^	4.00 ± 0.64**a**^**3**^**b**^**3**^
	7	0.27 ± 0.45	3.50 ± 0.51**a**^**3**^	4.27 ± 0.78**a**^**3**^**b**^**3**^

Statistical evaluation: The Kruskall-Wallis test (between columns), ANOVA test (within columns); **b**^**1**^, **b**^**2**^, **b**^**3**^ - P < 0.05, 0.01, 0.001 vs. asoxime-treated group; **c**^**1**^, **c**^**2**^ - P < 0.05, 0.01 vs. 1^st^ day.

Hepatic injuries perceived in rats treated exposed acutely to 0.5LD_50_ of each oxime were ranged from vacuolar degeneration to necrosis of isolated hepatocytes, which were associated with focal, moderate haemorrhage. As the term implies, such hepatic injuries were the foremost in the central lobular areas. In rats treated by asoxime and K027, one day after treatment, intracellular oedema and the occurrence of individual intracytoplasmic vacuoles are the only signs of cellular alteration. In the sinusoidal and in the perisinusoidal spaces discreet oedema, hyperaemia, haemorrhages and inflammatory cells were seen. In these areas, Kupffer’s cells were enlarged and allocated on the sinusoidal surface of the endothelial cells. Based on these mild or moderate morphologic alterations, the HDS values of asoxime- or K027-treated groups were in the range of 1.90 ± 0.61 to 2.10 ± 0.76. In these groups of animals, the focal necrosis of hepatocytes was observed, too. Focal necrosis consisted of discrete areas of the hepatic necrotic lobule. The necrotic areas were frequently small, involving only three or four cells. In addition to the necrotic hepatocytes, a small number of mononuclear inflammatory cells are frequently found in the lesion. Small necrotic foci were observed throughout the whole hepatic tissue. No fibrosis was observed. On the other hand, the highest HDS values were established in the group’s rats treated by obidoxime, K075 or K048 (P < 0.001 vs. control or asoxime-treated rats). Moreover, in these groups of animals, the presence of severe vacuolar or parenchymal degeneration, diffuse hyperaemia, large haemorrhagic foci and tissue infiltration with neutrophils, lymphocytes, plasma cells and macrophages were accompanied by central lobular necrosis. In addition, macronodular vacuolization of hepatocytes was expressed in majority hepatic sections. These morphological changes of the hepatic cytoplasm were commonly associated with the pyknotic nucleus. In some areas of the same hepatic tissue section, central lobular necrosis could be seen. According to these facts, the highest HDS value was in the K075-treated animals sacrificed 24 hrs after treatment (3.06 ± 0.78). In the K048-treated group, HDS value was a little less, but also significantly different from those established in the asoxime-treated or control rats (P < 0.001). Similar differences were noticed in these groups of rats sacrificed at the end of the study (Fig. 5b,c). It was found that the time depends HDS values were less significant in the asoxime-treated, K027-treated or K048-treated rats (P < 0.01) (Table 3).

When rats were exposed to extremely high dose, 1.0LD_50_ of each oxime, hyaline degeneration accompanied by diffuse haemorrhages, as well as massive necrosis in all treated animals were presented. When adjacent lobules affected, the necrosis was involved in large areas and all or nearly all hepatocytes in these areas. However, the enormous destroying of the lobular structure was surrounded by variably sized nodules with a normal lobular structure. In the early period, HDS values were the greatest in the K048-treated or K075-treated rats (4.33 ± 0.53 and 4.27 ± 0.78, respectively), and noticeably higher in comparison to the asoxime-treated rats (Fig. 5d) or the control rats (P < 0.001). Within a week, the alterations were spread to the entire liver tissue, so HDS values were higher compared to those which were visible on the first day, but with highest differences only in the K027-treated (Fig. 5e) or K048-treated rats (Fig. 5f) (P < 0.05) (Table 3).

### Pathohistological and semiquantitative analysis of experimental animal’s splenic alterations

**Figure 6:**
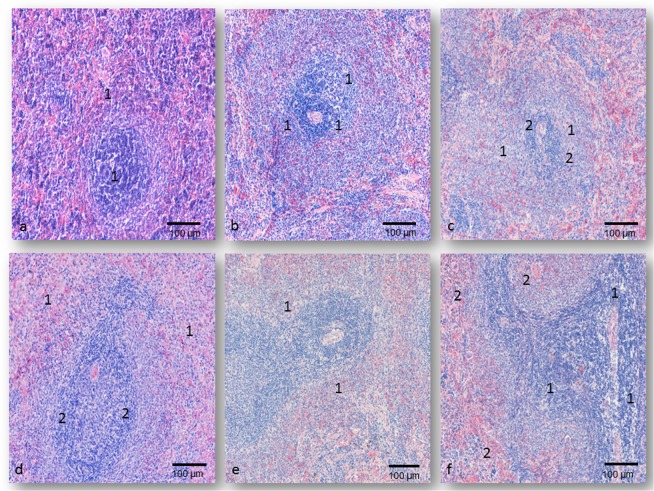
Splenic micrographs of rats on day 7 following oximes’ application; H&E staining; magnification at 200×; (**a**) The control group, normal histological structure of the splenic tissue - **1**; (**b**) The K048-treated group (0.5LD_50_ *im*), focal hemorrhages in the red pulp - **1**; (**c**) The K075-treated group (0.5LD_50_ *im*), diffuse hemorrhages - **1**, and accumulation of the plasma cells in the red pulp - **2**; (**d**) The asoxime-treated group (1.0LD_50_ *im*), moderate oedema and hyperemia in the trabeculae - **1**, diffuse lymphocytes depletion - **2**; (**e**) The K048-treated group (1.0LD_50_ *im*), inverted splenic architectures - **1**; (**f**) The K075-treated group (1.0LD_50_ *im*), necrotic foci - **1**, focal and massive hemorrhages, moderate fibrosis in the red pulp - **2**.

Splenic histological structure of rats treated by 0.1LD_50_ of each oxime was almost the same as those observed in the control group (Fig. 6a). In several tissue sections in the sinusoids of the red pulp mild oedema and hyperemia were seen. The size, shape and presence of the macrophages, lymphocytes and plasma cells were normal. The histological architectures of the white pulp were normal, too. The frequency of the vascular changes was the highest in the K075-treated rats. The difference between these SDS values, and those that were established in the other experimental groups was not expressed (Table 4).

**Table 4:** The influence of various oximes (0.1LD_50_, 0.5LD_50_ or 1.0LD_50_) on the splenic injury (splenic damage score, SDS) on day 1 and 7 following application.

Treatments	Days after treatment	SDS (5 spleens/group x 6 specimens/spleen) $\bar{{\boldsymbol{\times }}}$ ± S.D.		
		0.1LD_50_	0.5LD_50_	1.0LD_50_
control	1	0.10 ± 0.31	0.10 ± 0.31	0.10 ± 0.31
	7	0.17 ± 0.38	0.17 ± 0.38	0.17 ± 0.38
asoxime	1	0.20 ± 0.41	1.70 ± 0.70**a**^**3**^	2.10 ± 0.76**a**^**3**^
	7	0.20 ± 0.41	2.40 ± 0.50**a**^**3**^**c**^**2**^	3.00 ± 0.64**a**^**3**^**c**^**2**^
obidoxime	1	0.20 ± 0.41	2.03 ± 0.61**a**^**3**^	2.10 ± 0.65 **a**^**3**^
	7	0.27 ± 0.45	2.77 ± 0.69**a**^**3**^**c**^**1**^	3.03 ± 0.69**a**^**3**^**b**^**1**^**c**^**2**^
K027	1	0.17 ± 0.38	2.10 ± 0.76**a**^**3**^	3.43 ± 0.50**a**^**3**^**b**^**2**^
	7	0.23 ± 0.43	2.87 ± 0.82**a**^**3**^**c**^**2**^	4.17 ± 0.70**a**^**3**^**b**^**3**^**c**^**2**^
K048	1	0.23 ± 0.43	2.73 ± 0.87**a**^**3**^**b**^**3**^	4.20 ± 0.85**a**^**3**^**b**^**3**^
	7	0.23 ± 0.43	3.27 ± 0.69**a**^**3**^**b**^**3**^**c**^**2**^	4.53 ± 0.51**a**^**3**^**b**^**3**^
K075	1	0.30 ± 0.47	3.03 ± 0.76 **a**^**3**^**b**^**3**^	4.10 ± 0.84**a**^**3**^ **b**^**3**^
	7	0.33 ± 0.48	4.13 ± 0.78**a**^**3**^**b**^**3**^**c**^**3**^	4.33 ± 0.71**a**^**3**^ **b**^**3**^

Statistical evaluation: The Kruskall-Wallis test (between columns), ANOVA test (within columns); **b**^**1**^, **b**^**2**^, **b**^**3**^ - P < 0.05, 0.01, 0.001 vs. asoxime-treated group; **c**^**1**^, **c**^**2**^, **c**^**3**^ - P < 0.05, 0.01, 0.001 vs. 1^st^ day.

The first observable spleen alterations were detected in the rats treated by 0.5LD_50_ of each oxime. Histologically, a mild to severe depletion of lymphocytes in the white pulp was observed. The lymphocyte depletion was the most intensive in the periarteriolar lymphocyte sheets (PALS), so-called T lymphocytes areas. The germinal centres without changes were persisted in the PALS of the lymphatic nodules. In several tissue sections, moderate dilatations of the central arterioles were seen, too. In the outer part of the PALS, normal B lymphocytes and a larger number of the plasma cells were presented. These histological alterations were associated with focal, moderate vascular changes of the red pulp and trabeculae, such as oedema, hyperaemia and haemorrhage. Also, observed microscopic findings were the most intensive in the inner parts of the spleen tissue, especially in the K048 and in the K-075-treated animals, sacrificed on day 1 and day 7 (Fig. 6b,c). The highest SDS value of 4.13 ± 0.78 was in the K075-treated rats, sacrificed on day 7 (P < 0.001 vs. the 1^st^ day) (Table 4).

Described splenic injuries were also seen in the rats treated by 1.0LD_50_ of each oxime, but their intensity was more frequent. The prominent depletion of lymphocytes was presented, too. Depending on the time of sacrification, these affected areas were containing a small or large number of the plasma cells, as the final refuge of lymphoid cells. Intensive vacuolisation of the endothelial with pyknotic nuclei could be seen. Diffuse haemorrhages which were associated with prominent fibrosis were presented in the red pulp of rats treated by K027, K048 (Fig. 6e) or K075 (Fig. 6f), during the whole study period. Their frequency was certainly higher than in asoxime-treated rats (Fig. 6d) or in the control rats (P < 0.001). On the other hand, the SDS values of each time of sacrification were statistically different in the asoxime- and obidoxime-treated rats (P < 0.001) (Table 4).

## Discussion

Recognizing the exact mechanisms of oximes’ toxicity is the basic stage for not only in improving their safety testing but also for an accurate risk assessment based on these adverse effects. Earlier researches were mainly focused on the oximes’ therapeutic efficacy, while preclinical studies of the safety of oxime usage were poorly provided^27^. In many cases, preclinical tests on animals serve to accurately predict the adverse effects of new drug candidates in humans and the overall risk assessment of their exposure. Therefore, our study was based on defining the basic mechanisms of oximes’ toxicity, using standard safety testing, without imposing new preclinical methods or techniques. Further improving oximes’ safety testing will allow for a successful risk assessment based upon mechanisms of their toxicity.

In accordance with this challenge, the present study aimed to prove our hypothesis that exposure to a toxic dose of different oxime induced visceral tissues injury as well. Therefore, this study was also conducted entirely with male rats and followed our 7-day protocol, and gut, lung, liver and spleen injury was confirmed by histopathology and semiquantitative analyses. Our previous study has confirmed that exposure to low, injury-free oxime concentration, which was unable to cause muscle cells’ damage. Further increase in their doses leads to different muscular tissue damages due to their potent cytotoxic and pro-inflammatory effect^25^. Several investigators confirmed this statement *in vitro*^18,19^, but not so far *in vivo*^18,19^. Namely, K-oximes showed better antidotal efficacy than HI-6 at doses of 5% or 25% of their LD_50_^28^. Unfortunately, their high acute toxicity is a limiting factor for potential therapeutic usage. Accordingly, the primary adverse effects were observed in rats 24 hrs after K027, HI-6 and obidoxime application at a dose of 50% LD_50_^29^. Previous studies indicate that early toxic injury and autoxidative cellular injury are probably common mechanisms of oxime-induced cellular damage^30,31^. The presence of oximes, in high doses, might interfere with mitochondrial metabolism, eventually contributing to cell death and tissue necrosis, but it may also cause the development of reactive oxygen species (ROS) either directly or indirectly, resulting in cell homeostasis disturbance^32^.

The first signs of disruption of cell homeostasis in our experiments were verified by semiquantitative histopathological analysis 1 day after treatment with 0.5LD_50_ of different oxime, i.e. large areas of gastric, pulmonary, hepatic and spleen tissue had various tiny vacuoles and pale cytoplasm shape (mean GDS, PDS, HDS and SDS were in the range of 1.40 to 3.53), and discrete amounts of inflammatory cells were also identified. The most distinguished increase of GDS, PDS, HDS and SDS were detected in each tissue sample treated by 1.0LD_50_ of K048 and K075 (the mean GDS, PDS, HDS and SDS ranged from 3.60 to 4.36, P < 0.001 vs. asoxime-treated groups). At the highest concentrations of K048 and K075, the initial event is tissue necrosis, which mainly results in the destruction of cell membranes, leading to enhanced autophagocytosis and cells’ disappearance^33^. Many reactive intermediates of toxic drug doses are electrophiles, free radicals, or free-radical generators, which may potentiate the toxicity of tissue oxygen, depleting intracellular glutathione and biological antioxidants^34^. Moreover, the intensive release of reactive oxygen species (ROS) and free radicals that damage both DNA the cytoplasmic organelles and endoplasmic reticulum. The endoplasmic reticulum plays an important role within the intracellular quality control and sensitivity to oxidative stress^34,35^, and its stress is mainly related to the activation of apoptosis^36^. Several authors confirmed that ROS may act as messengers between the oxidative stress and endoplasmic reticulum stress^37,38^. During oxidative stress, mitochondrial dysfunction leads to the impaired production of ATP and a further increase in ROS production^39^.

On the other hand, neutrophils excrete a huge amount of proinflammatory molecules (i.e. cytokines, chemokine, and growth factors), and create a suitable microenvironment for monocytes and macrophages collecting^40–44^. Immediately after drug-induced tissue injuries, pro-inflammatory macrophages conduce to cell lysis and stimulate tissue proliferation, then a few days after toxic injury anti-inflammatory macrophages diminish the inflammatory reaction and stimulate tissue repair process^45^. The tissue regeneration process activated by low doses of the drug, while their high doses suppress compensatory tissue repair leading to improvement of tissue toxic injury^46,47^. In our study, after a period of 1 week, the prominent toxic tissue alterations were noticed in all visceral tissues following treatment with 0.5LD_50_ of obidoxime, K027, K048 and K075 (the mean PDS, GDS, HDS and SDS ranged from 2.63 to 4.13). Also, these tissue damage scores were the lowest at the end of the study only in the asoxime-treated group (the mean PDS, GDS, HDS and SDS ranged from 2.33 to 2.67). Afterwards, estimated PDS, GDS, HDS and SDS were enhanced after treatment by 1.0LD_50_ of asoxime but these scores were significantly lower in comparison to other oxime-treated groups (P < 0.001). The visceral tissues injuries with the highest were noticed after a week period in rats treated by 1.0LD_50_ of K048 and K075. The maximal calculated values of PDS, GDS, HDS and SDS were in the range of 4.13 to 4.53. These data, as well as ours previously published results^25^, confirm that the toxicity of oximes is time-dependent. Then, depending on the applied dose, the acute toxic tissue injury is associated with oximes-induced general toxicity.

Based on the obtained data within this study, as well in the previous one^25^, we can assume that 10% LD_50_ of all tested oximes is safe. In the case of asoxime and K027, also 50% LD_50_ seems to be still safe. So that, dosage 10% LD_50_ could be in future considered as appropriate treatment dosage which will have no adverse effect on treated organism regardless of oxime choice. Higher dosage is a question of selected oxime reactivator. As resulted from the obtained data, asoxime seems to be the safest oxime reactivator tested within these studies. This fact is in very good agreement with its acute toxicity data.

Briefly, our research confirmed that each oxime given by a single, high, unitary dose regimen, has an adverse effect not only on the main visceral tissues but on the whole rat as well. In addition, our results provided certain evidence of beyond the adverse effects on the target tissues and general health condition of rats after exposure to different oximes. Moreover, basic histopathology analyses can help to establish toxicological dose-response attitude in a preclinical safety assessing for newel oximes. Therefore, our data can subserve for the choice of dose in further subacute, subchronic or chronic evaluation. The application of these results can be used in non-clinical safety evaluation and the development of new oximes in order increasing the ability to test and improve the identification of the first signs of oxime-induced target tissue adverse effects. Finally, these results together with other preclinical data have a crucial contribution in the examination of general safety and risk assessment of newly developed oximes.

## Methods

### Used chemicals

Five AChE reactivators - two standard oximes (asoxime, obidoxime) and three experimental K-oximes (K027, K048, and K075) (Fig. 2) were used for *in vivo* experiments. All three experimental K-oximes were chosen for this evaluation according to previously published data^48^. Their detailed synthetic preparations have been described earlier in the literature^49–51^. Prior to the study, their purity was tested using the standard analytical methods^52,53^.

### Experimental animals

This study was performed on adult male Wistar rats (8 weeks old with body weight 180–220 g) breed at the Institute of Medical Research, Military Medical Academy, Belgrade, Serbia. Plastic cages (Macrolon cage type 4, BIOSCAPE, Germany) filled with sawdust (VERSELE-LAGA, Belgium) were used for the accommodation of rats. Ambient conditions, monitored by the central computerized system, were set up in the following range: temperature 22 ± 2 °C, relative humidity 55 ± 15%, 15–20 air change/h, and 12 hrs light/dark cycles. Animals had free access to filtered water and commercial pellets for rats, produced by Veterinary Institute Subotica, Serbia.

The Ethics Committee for Experiments on Animals, of the Military Medical Academy, Belgrade, Serbia approved (i) animal welfare procedures and study design (No. 282-12/2002); (ii) experimental protocol was performed according to the Guidelines for Animal Welfare of the Ethics Committee for Experiments on Animals of the Military Medical Academy, Belgrade, Serbia (No. 323-07-04943/2014-05/1), and the National Guidelines for Animal Welfare, Belgrade, Serbia (No. 41/2009).

### Acute toxicity

Each oxime was preliminarily tested in animals using standard experimental procedure published earlier in the literature^54^. Thereafter, each oxime was used in the present study at a single dose of 0.1LD_50_, 0.5LD_50_ and 1.0LD_50_.

### Experimental design

Wistar rats were divided into sixteen experimental groups as shown in Table 5.

**Table 5:** The experimental design and oximes’ doses.

Experimental group	Treatments	Total number of animals/group (n)	Dose (mg/kg *im*)
1.	control	10	0.9% NaCl (1 ml/kg)
2.	asoxime	10	0.1LD_50_ (63 mg/kg *im*)
3.	asoxime	10	0.5LD_50_ (313 mg/kg *im*)
4.	asoxime	20	1.0LD_50_ (626 mg/kg *im*)
5.	obidoxime	10	0.1LD_50_ (16 mg/kg *im*)
6.	obidoxime	10	0.5LD_50_ (82 mg/kg *im*)
7.	obidoxime	20	1.0LD_50_ (164 mg/kg *im*)
8.	K027	10	0.1LD_50_ (66 mg/kg *im*)
9.	K027	10	0.5LD_50_ (332 mg/kg *im*)
10.	K027	20	1.0LD_50_ (664 mg/kg *im*)
11.	K048	10	0.1LD_50_ (23 mg/kg *im*)
12.	K048	10	0.5LD_50_ (115 mg/kg *im*)
13.	K048	20	1.0LD_50_ (230 mg/kg *im*)
14.	K075	10	0.1LD_50_ (8 mg/kg *im*)
15.	K075	10	0.5LD_50_ (41 mg/kg *im*)
16.	K075	20	1.0LD_50_ (82 mg/kg *im*)

Immediately before the intramuscular application, each oxime is dissolved in a freshly prepared solution of normal saline (0.9% sodium chloride in distilled water). A single injection of each oxime was applied in lateral thigh muscle of the right leg^55^. The general health condition of animals was monitored daily throughout the study, while a standard post-mortem examination was performed for 7 days.

The selected doses for this histopathological study was described in our previously published studies^25^. As we mentioned in those studies, the selection of dose levels for careful histopathology analysis was primarily based on the results of acute toxicity testing^26^. The following 4 dose levels were selected to evaluate dose-dependent acute toxicity for different tissues at two different time intervals: control group; low dose group (5–10% of LD_50_), medium-dose group (≈50% of LD_50_) and high dose group (≈1.0 LD_50_). Specifically, in line with the OECD GLP-compliant guidelines, at the end of the acute toxicity tests, a pathohistological analysis is required to assess the safety of the chemicals being investigated^56–58^.

### Histopathological procedure

Five animals from each experimental group were sacrificed, using light ether anaesthesia, on the 1^st^ and 7^th^ after the applied treatments^25,59^.

At autopsy, different tissue samples (lung, stomach, liver, and spleen) of each rat were fixed (10% neutral-buffered formalin over 48 hrs) and prepared for further standard histopathological analysis. Tissue’s dehydration was carried out by immersing specimens in a series of ethanol solutions of increasing concentration until pure, water-free alcohol is reached. Ethanol is miscible with water in all proportions so that the water in the specimen was progressively replaced by the alcohol. A series of increasing concentrations was used to avoid excessive distortion of the tissue. A typical dehydration process for specimens, not more than 4 μm thick was: 70% ethanol for 15 min, 90% ethanol for 15 min, 100% ethanol two times for 15 min, 100% ethanol for 30 min and 100% ethanol for 45 min, respectively. At this point, all but a tiny residue of tightly bound molecular water were removed from the specimen.

After dehydration, each tissue sample was embedded into melted paraffin wax, the block was mounted on a microtome and cut into thin slices (2 µm). The slices were affixed to microscope slides at which point the wax was removed with a solvent and the tissue slices attached to the slides are rehydrated and were ready for staining. Finally, after the application of hematoxylin, followed by a rinse in a weak acid solution to remove excess staining, each tissue slice was counterstained with eosin^60^.

For detail histopathological and semiquantitative analyses, Olympus BKS-43 (OLYMPUS, Japan) with a digital camera and Cell D software (MUNSTER, Germany) were used. Complete histopathological and semiquantitative analyses were performed by pathologists blind of the treatment groups.

### Semiquantitative analysis

The intensity of degenerative and vascular lesions in the lungs, stomach, liver and spleen were scored and counted through a light microscope in 30 tissues’ slices per group (i.e., five tissues from each experimental group and six slices from each tissue) with a magnification of 200x according to previously published literature^25,61–67^.

As shown in Table 6, the severity grades were expressed as gastric damage score (GDS), pulmonary damage score (PDS), hepatic damage score (HDS), and splenic damage score (SDS). Besides, the precise procedures of their calculating are shown in Tables 1–4.

**Table 6:** The classification of visceral tissues injuries - gastric damage score (GDS), pulmonary damage score (PDS), hepatic damage score (HDS), and splenic damage score (SDS).

Degree	Description
0	Histological components without changes.
1	Stomach: Single desquamated epithelial cells. Individual inflammatory cells in the *tunica submucosa*.
	Lung: A few alveoli were filled with desquamated epithelial cells and single alveolar macrophages. Small foci of the perivascular inflammatory cell infiltrate in the pulmonary interstitium.
	Liver: Individual hepatocytes with pronounced oedema. Discreetly expanded blood vessels surrounded by single inflammatory cells.
	Spleen: The red pulp with oedema and hyperemia. Perivascular presence of the macrophages, lymphocytes and plasma cells.
2	Stomach: Small groups of gastric pits filled with desquamated epithelial cells and mucus. Discrete transmular oedema and hyperaemia. Inflammatory cell infiltrates in the *tunica mucosa* and *tunica submucosa*.
	Lung: Minority alveoli filled with desquamated epithelial cells and seromucous fluid. Focal perivascular interstitial oedema and hyperaemia. A large number of inflammatory cells infiltrates in the pulmonary parenchyma.
	Liver: Micronodular vacuolisation of the hepatocytes with the normal nucleus. Focal oedema, hyperemia and haemorrhage of the sinusoids. A various number of perivascular inflammatory cell infiltrates.
	Spleen: Focal depletion of lymphocytes in the periarteriolar lymphatic sheets, and normal germinal centres. Dilatation of the central arterioles. A various number of macrophages and the plasma cells.
3	Stomach: Superficial defects in the *tunica mucosa*. Intensive transmular oedema and hyperaemia. Focal haemorrhage and inflammatory cells in all stomach’ layers.
	Lung: Majority of the alveolar epithelial cells and endothelial cells are damaged. Intensive interstitial oedema and hyperaemia. Focal haemorrhage and accumulation of inflammatory cells in the pulmonary parenchyma.
	Liver: Macronodular vacuolization of the hepatocytes with the picnotic nucleus. Accumulation of inflammatory cells and focal haemorrhage.
	Spleen: Focal depletion of lymphocytes in the periarteriolar lymphatic sheets, and normal germinal centres. Dilatation of the central arterioles. A various number of the macrophages, lymphocytes and the plasma cells.
4	Stomach: Gastric ulcerations in the *tunica mucosa* and *tunica submucosa*. Massive haemorrhage and inflammatory cells infiltrate in the *tunica mucosa*.
	Lung: Massive intra-alveolar and interstitial oedema. Alveolar space filled with damaged epithelial cells and scavenger macrophages. Massive haemorrhage foci and inflammatory cells infiltrate in the pulmonary parenchyma. and *tunica submucosa*. Gastric pits enlarged and filled with necrotic debris.
	Liver: Central lobular necrosis, diffuse haemorrhage and inflammatory cells infiltrate.
	Spleen: Intensive depletion of all lymphocytes in the lymphatic nodules. Massive haemorrhages, and a large number of phagocytic cells.
5	Tissue necrosis.

### Statistical analysis

For statistical evaluation, commercial statistical software (Stat for Windows, R.7, STAT SOFT, INC., USA, 2008) was used. All results shown in tables were expressed as the mean ($\bar{{\rm{x}}}$) ± the standard deviation (S.D.). Firstly, the normality of the data distribution was evaluated using a Kolmogorov-Smirnov test. Then, using these results the variances in the damage scores i.e. PDS, GDS, HDS and SDS, within and between the sets under discussion were evaluated through the nonparametric Kruskal-Wallis test (ANOVA for multiple evaluations). All variances were approximated at minimum P < 0.05 level of statistical significance.

### Ehhthical approval

The Ethics Committee for Experiments on Animals, of the Military Medical Academy, Belgrade, Serbia approved (i) animal welfare procedures and study design (No. 282-12/2002); (ii) experimental protocol was performed according to the Guidelines for Animal Welfare of the Ethics Committee for Experiments on Animals of the Military Medical Academy, Belgrade, Serbia (No. 323-07-04943/2014-05/1), and the National Guidelines for Animal Welfare, Belgrade, Serbia (No. 41/2009).
